# Characterization of Antimicrobial Resistance in *Serratia* spp. and *Citrobacter* spp. Isolates from Companion Animals in Japan: Nosocomial Dissemination of Extended-Spectrum Cephalosporin-Resistant *Citrobacter freundii*

**DOI:** 10.3390/microorganisms7030064

**Published:** 2019-02-28

**Authors:** Kazuki Harada, Takae Shimizu, Hiroichi Ozaki, Yui Kimura, Tadashi Miyamoto, Yuzo Tsuyuki

**Affiliations:** 1 Department of Veterinary Internal Medicine, Tottori University, Tottori 680-8553, Japan; takae.shimizu@ani-com.com; 2 Anicom Specialty Medical Institute Inc., Kanagawa 231-0033, Japan; 3 Department of Veterinary Microbiology, Tottori University, Tottori 680-8553, Japan; ikazo-h@tottori-u.ac.jp; 4 Miyamoto Animal Hospital, Yamaguchi 753-0851, Japan; v008tm@yamaguchi-u.ac.jp (Y.K.); miya629@c-able.ne.jp (T.M.); 5 Sanritsu Zelkova Laboratory, Tokyo 135-0011, Japan; y-tsuyuki@san-g.com

**Keywords:** *Serratia* spp., *Citrobacter* spp., companion animals, extended-spectrum cephalosporin resistance, nosocomial dissemination

## Abstract

In many countries including Japan, the status of emerging antimicrobial resistance among *Serratia* spp. and *Citrobacter* spp. in companion animals remains unknown because these genera are rarely isolated from animals. In this study, 30 *Serratia* spp. and 23 *Citrobacter* spp. isolates from companion animals underwent susceptibility testing for 10 antimicrobials. Phenotypic and genetic approaches were used to identify the mechanisms of extended-spectrum cephalosporins (ESC). Subsequently, ESC-resistant *Citrobacter* spp. strains underwent multilocus sequence typing and pulsed-field gel electrophoresis (PFGE). A significantly higher rate (34.8%) of ESC resistance was observed in *Citrobacter* spp. isolates than in *Serratia* spp. isolates (0%). ESC resistance was detected in five *C. freundii* strains, two *C. portucalensis* strains, and one *C. koseri* strain. All of the ESC-resistant *Citrobacter* spp. strains harbored CMY-type and/or DHA-type AmpC β-lactamases. Three *C.* *freundii* strains harbored the CTX-M-3-type extended-spectrum β-lactamases. Notably, the three *bla*CTX-3-producing and two *bla*CMY-117-bearing *C. freundii* strains (obtained from different patients in one hospital) had the same sequence type (ST156 and ST18, respectively) and similar PFGE profiles. We believe that ESC-resistant *Citrobacter* spp. are important nosocomial pathogens in veterinary medicine. Therefore, infection control in animal hospitals is essential to prevent dissemination of these resistant pathogens.

## 1. Introduction

The genera of *Serratia* and *Citrobacter*, belonging to the *Enterobacteriaceae* family, are opportunistic nosocomial pathogens and cause a wide spectrum of human infections [1,2]. In companion animals, these genera are also rarely associated with infections such as pneumonitis, urinary tract infection, myocarditis, septicemia, and intravenous catheter site infection [3]. The *Citrobacter* genus previously comprised 11 species [4], but recently, two new species (i.e., *C. pasteurii* and *C. portucalensis*) have been proposed [5,6]. *Serratia marcescens* and *Citrobacter freundii* are the most important species in each genus, medically speaking [7,8]. However, the species distribution in these genera has not yet been elucidated in veterinary medicine.

The emergence of multidrug resistance—notably, resistance to extended-spectrum cephalosporins (ESC)—among *Serratia* spp. and *Citrobacter* spp. is a worldwide concern to human medicine [1]. Together with *Pseudomonas* spp., *Acinetobacter* spp., and *Enterobacter* spp., these genera are hospital-acquired Gram-negative bacilli which can easily develop antimicrobial resistance and are often grouped as the SPACE organisms [9]. ESC resistance in these bacteria is usually caused by overproduction of AmpC β-lactamases, secondary to the derepression of a chromosomal gene or acquisition of a transferable AmpC β-lactamase [9]. Additionally, extended-spectrum β-lactamases (ESBLs) and carbapenemases have been identified in *Serratia* spp. and *Citrobacter* spp. [1,10], which exacerbates ESC resistance. However, in many countries including Japan, the status of emerging antimicrobial resistance among *Serratia* spp. and *Citrobacter* spp. in companion animals remains unknown. 

Therefore, we performed an epidemiological investigation of the predominance of antimicrobial resistance and provide molecular characterization of ESC resistance among *Serratia* spp. and *Citrobacter* spp. isolates recovered from clinical specimens of dogs and cats in Japan.

## 2. Materials and Methods

We evaluated a total of 53 clinical isolates including 30 *Serratia* spp. and 23 *Citrobacter* spp. isolates collected from dogs (*n* = 36) and cats (*n* = 17) housed by different owners who visited veterinary hospitals between 2012 and 2016. Table S1 shows the details of the isolates used in this study, including the specific locations of hospitals and isolation sites. Specimens were isolated using sterile cotton swabs from various anatomical sites, identified as sites of bacterial infection by many clinical veterinarians in 15 prefectures in Japan and submitted to Tottori University and Sanritsu Zelkova Laboratory for analysis. All confirmed isolates were stored at −80 °C in 10% skim milk.

Bacterial species identification was determined based on growth conditions on CHROMagar orientation medium (Nippon Becton Dickinson and Company, Ltd., Tokyo, Japan) and by using the API 20E kit (SYSMEX bioMérieux Co., Ltd., Tokyo, Japan), MicroScan WalkAway (Beckman Coulter, Inc., Tokyo, Japan), and MALDI-TOF MS with a Bruker MALDI Biotyper system (Bruker Daltonik, Bremen, Germany). Additionally, the species of *Citrobacter* spp. isolates were confirmed based on the phylogeny of the *recN* (DNA repair protein) gene, as described previously [11]. Briefly, PCR amplification and further sequencing of *recN* genes were performed. Then, we constructed a phylogenetic tree based on the *recN* sequences of our strains and type strains of each species using MEGA version 7.0.18 [12] using the neighbor-joining (NJ) method [13]. The genetic distance among these strains was calculated using the Kimura two-parameter model [14].

We determined the susceptibilities of these bacterial species to amoxicillin–clavulanic acid (ACV, Sigma-Aldrich Co. LLC., Tokyo, Japan), cephalothin (CPL, Sigma-Aldrich), cefmetazole (CMZ, Sigma-Aldrich), cefotaxime (CTX, Wako Pure Chemical Industries, Ltd., Osaka, Japan), meropenem (Wako Pure Chemical), tetracycline (TET, Wako Pure Chemical), amikacin (Sigma-Aldrich), chloramphenicol (CHL, Wako Pure Chemical), trimethoprim/sulfamethoxazole (TMS, Wako Pure Chemical), and ciprofloxacin (CIP, Wako Pure Chemical). We used the agar dilution method to perform susceptibility testing in accordance with the Clinical and Laboratory Standards Institute (CLSI) guidelines [15]. The susceptibility results were interpreted in relation to the CLSI guideline criteria [16]. *Escherichia coli* ATCC 25922 was used as a control strain. ESC-resistant (i.e., minimum inhibitory concentration (MIC) for CTX of ≥ 4 µg/mL) strains were screened for ESBLs using the double-disc synergy test with CTX, ceftazidime, cefepime, and ACV disks on Mueller–Hinton agar plates with or without 200 µg/mL cloxacillin [17]. Additionally, ESC-resistant isolates without the synergistic effect of clavulanate and with inhibition zones enhanced by cloxacillin were classified as organisms overexpressing AmpC β-lactamase [18]. 

All ESC-resistant strains were screened for class A β-lactamase genes (i.e., *bla*TEM and *bla*SHV) and acquired AmpC β-lactamase (qAmpC) genes (i.e., the ACC, FOX, MOX, DHA, CIT, and EBC groups) on PCR as previously described [19,20]. The amplified products underwent bidirectional sequencing using specific primers [19,21]. In ESBL-positive strains, multiplex PCR was used to detect the CTX-M-type β-lactamase genes [22]. For the positive isolates, genes were amplified and sequenced to distinguish CTX-M subtypes using group-specific PCR primers [19]. A previous conjugation experiment [23] with slight modifications confirmed the transferability of ESBL genes. The *E. coli* DH5α strain (Thermo Fisher Scientific K.K., Tokyo, Japan) was used as a recipient, and transconjugants were selected on DHL-agar containing rifampicin (50 µg/mL) and CTX (2 µg/mL).

Pulsed-field gel electrophoresis (PFGE) was conducted on ESC-resistant *C. freundii* strains as previously described [2,24]. Bacterial DNA was digested with *Xba*I and *SfiI* and electrophoresed using CHEF-DR II (Bio-Rad Laboratories, Richmond, CA, USA). Then, PFGE profiles were digitized for analysis using GelCompar II (Applied Maths, Inc., Austin, TX, USA). Finally, multilocus sequence typing (MLST) with seven genes (i.e., *aspC*, *clpX*, *fadD*, *mdh*, *arcA*, *dnaG*, and *lysP*) was performed as previously described [25]. A new sequence type (ST) was submitted to the MLST website and new ST numbers were assigned.

## 3. Results and Discussion

**Figure 1:**
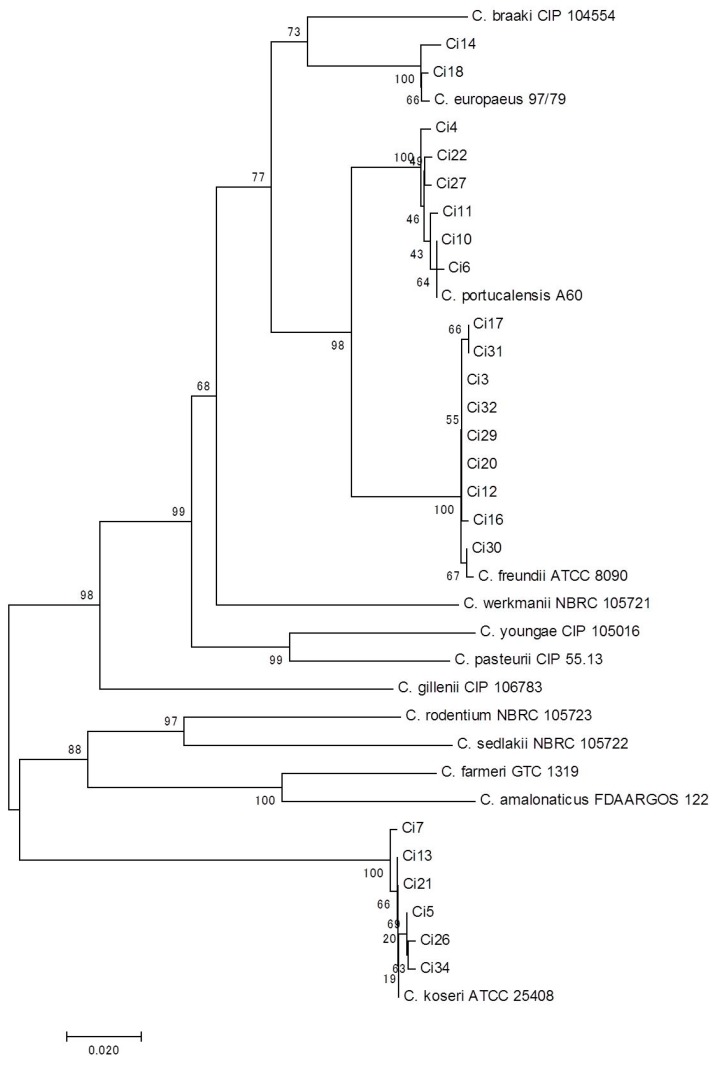
Neighbor-joining tree based on *recN* gene sequences from our data collection and type strains of *Citrobacter* species. Genetic distances were constructed using Kimura’s two-parameter method. Bootstrap values obtained after 1000 replicates are given at the nodes [11]. The corresponding GenBank/Patric accession numbers of type strains refer to the previous report by Ribeiro et al. [6].

Few studies have reported the species distribution and prevalence of antimicrobial resistance in the overall population of *Serratia* spp. and *Citrobacter* spp. clinical isolates from companion animals. The bacterial species of our collection were identified by several conventional methods and finally determined by MALDI-TOF MS (in *Serratia* spp.) and *recN* phylogeny (in *Citrobacter* spp.). We classified 30 *Serratia* spp. isolates into *S*. *marcescens* (*n* = 26), *S*. *liquefaciens* (*n* = 2), *S*. *fonticola* (*n* = 1), and *S. ureilytica* (*n* = 1); therefore, *S*. *marcescens* is most likely the major species of the genus in companion animals and in human medicine [4]. We conclusively determined 23 *Citrobacter* spp. isolates based on *recN* phylogeny as follows: *C*. *freundii* (*n* = 9), *C. koseri* (*n* = 6), *C*. *portucalensis* (*n* = 6), and C. *europaeus* (*n* = 2) (Figure 1). To the best of our knowledge, this is the first report on the isolation of *C. portucalensis* and *C. europaeus* strains from animals, although these species have been rarely reported in humans [26,27]. These findings imply the prevalence of several *Citrobacter* species in companion animals, in addition to *C. freundii*. We also found discrepancies in bacterial species between conventional phenotypic methods and the more reliable methods (i.e., MALDI TOF-MS and *recN* phylogeny) in several strains (Table S1); this suggests that phenotypic methods are limited in their ability to identify species of *Serratia* spp. and *Citrobacter* spp.

Table 1 shows the MIC distribution of the 10 tested antimicrobials in both genera. There were significant differences in overall resistance rates to six antimicrobials: *Serratia* spp. isolates exhibited higher rates of resistance to CPL (100%) and TET (86.7%), compared to the *Citrobacter* spp. isolates which exhibited higher rates of resistance to CTX (34.8%), CIP (26.1%), and TMS (17.4%). Previous work demonstrated higher rates of resistance to ACV (93.3%), CMZ (93.3%), CHL (46.7%), and CIP (43.3%) in *Enterobacter* spp. isolates from companion animals in Japan during 2003–2015 [28]. On the other hand, moderate rates of resistance to CIP (20.5% and 11.9%) were validated in *Pseudomonas* spp. and *Acinetobacter* spp. isolates from companion animals in Japan during the periods of 2003–2010 and 2012–2016, respectively [29,30], suggesting that SPACE organisms from companion animals have different antimicrobial resistance profiles by genus.

ESC resistance was identified in none of the *Serratia* spp. isolates and in 8 of 23 *Citrobacter* spp. isolates, namely, *C*. *freundii* (*n* = 5), *C. portucalensis* (*n* = 2), and *C*. *koseri* (*n* = 1) (Table 2). All ESC-resistant isolates had an MIC for MPM of ≤ 0.125 µg/mL, indicating that these isolates were negative for carbapenemase [31]. Of these ESC-resistant *Citrobacter* spp. strains, the three *C*. *freundii* strains harbored nontransferable *bla*CTX-M-3, which has previously been detected in *Enterobacteriaceae* in companion animals in Japan [28,32,33], as well as in France [34], South Korea [35], and China [36]. To the best of our knowledge, ours is the first report to detect *bla*CTX-M-3 among *Citrobacter* spp. isolates from companion animals, although Ewers et al. previously reported *C*. *freundii* isolates producing *bla*CTX-M-1 or *bla*SHV-12 in European countries [37]. The present study demonstrated ESBLs in 3 of 23 (13.0%) *Citrobacter* spp. isolates, comparable to previous work in companion animals in European countries (9/77, 11.7%) [37]. On the other hand, Kanamori et al. [2] previously detected ESBLs in 67 of 348 (19.3%) human isolates in Japan, but no evidence of *bla*CTX-M-3. Hence, it is likely that different types of ESBLs are prevalent in *Citrobacter* spp. isolates between companion animals and humans in Japan. 

We also found a prevalence of AmpC β-lactamases among eight ESC-resistant *Citrobacter* spp. strains. Of the qAmpC genes, CMY-family β-lactamases (previously reported in *Citrobacter* spp., accession numbers: NG_048788, NG_048875, NG_048832, and NZ_QRJT01000009) were detected in seven strains: five *C. freundii* and two *C. portucalensis* strains. On the other hand, *bla*DHA-1 was identified in one *C*. *portucalensis* and one *C*. *koseri* strain, as well as the other Gram-negative bacteria from companion animals [28,33,38]. In addition, each of the two strains of *C*. *freundii* and *C. portucalensis* were chromosomal AmpC hyperproducers, which can confer resistance to cephalosporins, including later-generation compounds and some penicillins [39,40]. Our data indicate that these AmpC-mediated resistance mechanisms, as well as ESBLs, play a role in the prevalence of ESC-resistant *Citrobacter* spp. strains in companion animals.

**Figure 2:**
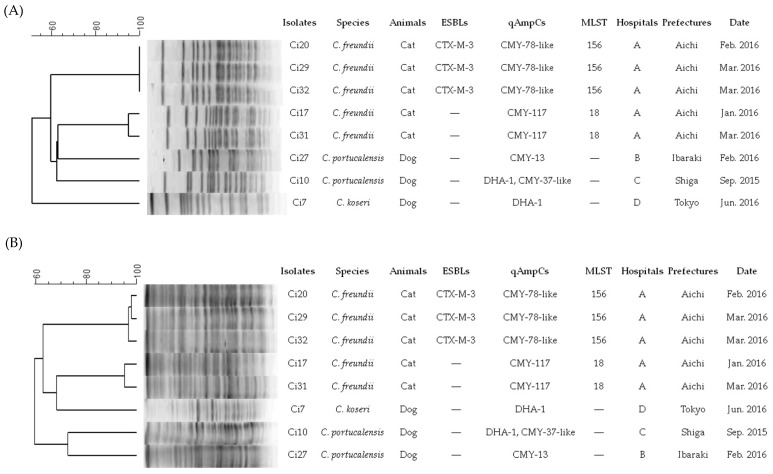
Pulsed-field gel electrophoresis (PFGE) profiles of eight ESC-producing *Citrobacter* strains digested with *XbaI* (**A**) and *SfiI* (**B**).

In the present study, we conducted MLST analysis for *C*. *freundii* isolates from animals for the first time. The three *bla*CTX-M-3-producing *C. freundii* strains (strains Ci20, Ci29, and Ci32) were assigned to ST156, which was the first ST identified in our study (Table 2). Additional analysis revealed a similar or identical antimicrobial susceptibility profiles and PFGE profiles of XbaI- and SfiI-digested genomic DNA among the three strains (Figure 2). In addition, the two CMY-117-bearing *C. freundii* strains (strains Ci17 and Ci31) were assigned to ST18, which was the previously identified ST in human-origin carbapenemase-producing *C. freundii* in Denmark [41], Spain [42], and Czech Republic [43], and had almost indistinguishable PFGE profiles. These ESC-resistant *C*. *freundii* strains were acquired from different animals in the same hospital, suggesting nosocomial infections. Similar findings have been observed in other ESBL-producing bacteria [28,33,38]. Therefore, infection control in hospitals is essential in preventing the dissemination of ESC-resistant *Citrobacter* spp. isolates among companion animals.

## 4. Conclusions

In conclusion, we described antimicrobial resistance, particularly ESC resistance, among *Serratia* spp. and *Citrobacter* spp. strains isolated from companion animals in Japan and established differences in the prevalence of antimicrobial resistance between those isolates. Moreover, we are the first to identify nosocomial dissemination of ESC-resistant *C*. *freundii* strains producing ESBLs or qAmpCs in companion animals. Although *Citrobacter* spp. are only rarely isolated from companion animals, these bacteria deserve continuous surveillance to determine the true risk of their antimicrobial resistance in veterinary and human medicine.

## Figures and Tables

**Table 1:** Minimum inhibitory concentration (MIC) distribution and resistance rates among *Serratia* spp. and *Citrobacter* spp. isolates from dogs and cats.

Agents	Genera (No. of Isolates)	MIC (µg/mL)															No. of Resistance (%)
		≤0.031	0.063	0.125	0.25	0.5	1	2	4	8	16	32	64	128	256	>256	
CPL	*Serratia* (30)															30	30 (100) *
	*Citrobacter* (23)							2	2	1		1	4	2	1	10	18 (78.3)
CMZ	*Serratia* (30)								2	9	10	5	4				4 (13.3)
	*Citrobacter* (23)					1	3	1		1	5	5	6	1			7 (30.4)
CTX	*Serratia* (30)			2	14	13	1										0 (0.0)
	*Citrobacter* (23)		3	6	6				1	1	1	2			1	2	8 (34.8) *
MPM	*Serratia* (30)	4	25	1													0 (0.0)
	*Citrobacter* (23)	19	3	1													0 (0.0)
TET	*Serratia* (30)						1	1		2	7	14	5				26 (86.7) *
	*Citrobacter* (23)					1	15	1	2			1	1	2			4 (17.4)
AMK	*Serratia* (30)					4	3	20	3								0 (0.0)
	*Citrobacter* (23)					2	5	13	2	1							0 (0.0)
CHL	*Serratia* (30)								2	14	10	2	1		1		4 (13.3)
	*Citrobacter* (23)									17	4	1		1			2 (8.7)
CIP	*Serratia* (30)	4	4	16	4		1	1									0 (0.0)
	*Citrobacter* (23)	6	2	2	3	1	1	2	2	3	1						6 (26.1) *
				0.125/0.063	0.25/0.125	0.5/0.25	1/0.5	2/1	4/2	8/4	8/16	32/16	64/32	128/64	256/128	>256/128	
ACV	*Serratia* (30)								1	3	1	2	15	8			25 (83.3)
	*Citrobacter* (23)							3	2		4	5	9				14 (60.9)
				≤0.03/0.59	0.06/1.19	0.13/2.38	0.25/4.75	0.5/9.5	1/19	2/38	4/76	8/152	16/304	32/608	64/1216	>64/1216	
TMS	*Serratia* (30)			4	5	18	3										0 (0.0)
	*Citrobacter* (23)			9	5	1	2	1		1		1				3	4 (17.4) *

CPL, cephalothin; CMZ, cefmetazole; CTX, cefotaxime; MPM, meropenem; TET, tetracycline; AMK, amikacin; CHL, chloramphenicol; CIP, ciprofloxacin; ACV, amoxicillin–clavulanic acid; TMS, trimethoprim–sulfamethoxazole. Vertical lines indicate breakpoints of each drug according to the Clinical and Laboratory Standards Institute guideline [10]. * Significant differences in resistance rates between *Serratia* spp. and *Citrobacter* spp. (*p* < 0.05).

**Table 2:** Characterization of eight extended-spectrum cephalosporin (ESC)-resistant *Citrobacter* spp. strains from dogs and cats.

Strain	Year	Host	Origin	ST	AmpC Overexpression	ESBLs	qAmpCs	Other β-lactamases	MIC (μg/mL)^b^									
									ACV	CPL	CMZ	CTX	MPM	TET	CHL	AMK	CIP	TMS
*C. freundii* (*n* = 5)																		
Ci17	2016	Cat	Urine	18	+		CMY-117	TEM-1	64/32	>256	64	32	0.063	1	16	2	8	8/152
Ci20	2016	Cat	Urine	156 *	-	CTX-M-3	CMY-78-like	TEM-1	16/8	>256	16	256	0.031	1	8	4	2	0.06/1.19
Ci29	2016	Cat	Urine	156 *	-	CTX-M-3	CMY-78-like	TEM-1	16/8	>256	8	>256	0.031	4	16	2	4	0.25/4.75
Ci31	2016	Cat	Urine	18	+		CMY-117		64/32	>256	64	16	0.063	128	8	1	8	>64/1216
Ci32	2016	Cat	Urine	156 *	-	CTX-M-3	CMY-78-like	TEM-1	32/16	>256	16	>256	0.031	32	16	8	8	2/38
*C. portucalensis* (*n* = 2)																		
Ci10	2015	Dog	Urine	NA	+		DHA-1, CMY-37-like		64/32	>256	64	8	0.125	128	128	2	16	>64/1216
Ci27	2016	Dog	Nasal	NA	+		CMY-13		64/32	>256	128	32	0.063	1	8	4	4	≤0.03/0.59
*C. koseri* (*n* = 1)																		
Ci7	2015	Dog	Urine	NA	NA		DHA-1		64/32	>256	64	4	0.015	1	8	1	0.25	0.5/9.5

NA, Not applicable. * ST156 was firstly identified in this study. ACV, amoxicillin–clavulanic acid; CPL, cephalothin; CMZ, cefmetazole; CTX, cefotaxime; MPM, meropenem; TET, tetracycline; CHL. Chloramphenicol; AMK, amikacin; CIP, ciprofloxacin; TMS, trimethoprim–sulfamethoxazole.

## Supplementary Materials

The following are available online at https://www.mdpi.com/2076-2607/7/3/64/s1, Table S1: The details of Serratia spp. and Citrobacter spp. isolates used in this study.
